# Association of Genetic polymorphisms of *EDN1* gene and Endothelin-1 level in patients with type 2 diabetes mellitus in the Jordanian population

**DOI:** 10.1016/j.heliyon.2023.e23676

**Published:** 2023-12-17

**Authors:** Ahmed O. Maslat, Omar M. Al-Mahmood, Nahla M. Al Khawaja, Ramadan Al-Shdefat

**Affiliations:** a Department of Biological Sciences, Faculty of Science, Yarmouk University, Jordan and Faculty of Pharmacy, Jadara University, Jordan; b Department of Biological Sciences, Faculty of Science, Yarmouk University, Jordan; c National Center for Diabetes, Endocrinology and Genetics, Jordan University, Jordan; d Faculty of Pharmacy, Jadara University, Jordan

**Keywords:** *Diabetes mellitus type 2*, *SNPs*, *Endothelin-1*, *Gene polymorphism*, *Single nucleotide polymorphism*, *Susceptibility markers*

## Abstract

Endothelin-1 (ET-1) is one of the most potent vasoconstrictors, encoded by the endothelin-1 *(EDN*1) gene. It has been shown to play an important role in different diseases including Diabetes Mellitus (DM). Various single nucleotide polymorphisms (SNPs) in the *EDN*1 gene are related to microvascular complications of type 2 diabetes mellitus (T2DM) such as retinopathy, neuropathy and nephropathy. This study aims to determine the association between two selected *EDN*1 gene polymorphisms (rs2071942 G > A, rs5370 G > T) and T2DM in the Jordanian population, also to measure the level of ET-1 in T2DM. The samples were collected from the National Center of Diabetes, Endocrinology, and Genetics- Amman, Jordan, including 97 patients with T2DM and 80 healthy individuals. PCR-RFLP was used for SNPs genotyping. ET-1 level was determined using IQELISA kits. The univariate analysis for both SNPs didn't show statistically signiﬁcant differences in the genotype or allele frequencies among T2DM cases as well as in controls. The same results were obtained regarding ET-1 concentration. The subgroup analysis by sex showed that the genotype and allelic frequencies of rs5370, rs2071942 G/A polymorphisms were not significantly different in males and females. Multivariate Analysis adjusted for various confounders didn't express statistical significance difference for occurrences of both SNPs. However, height and gender showed to be significant risk factors for occurrences of heterozygote alleles in both SNPs. On the other hand, the duration of diabetes has appeared to be related to the recessive allele in rs5370.

## 1 Introduction

Diabetes Mellitus (DM) is chronic hyperglycemia characterized by a deficiency in insulin secretion, insulin activity, or both [[1], [2], [3]]. DM has several types such as type 1 diabetes*,* type 2 diabetes, idiopathic diabetes, and gestational diabetes mellitus [4,5]. It exerts its effects on the endocrine system and on the vascular system. Diabetes complications include microvascular (nephropathy, retinopathy, and neuropathy) and macrovascular complications (heart disease, stroke, and peripheral arterial disease) [2,6]. Endothelial dysfunction may also contribute to the micro and macrovascular complications of diabetes [3,7]. Endothelial dysfunction leads to the upregulation of endothelin-1(ET-1) and this lead to changes in the blood vessel walls such as the formation of thrombosis and plaque. It was found that plasma ET-1 levels are elevated in patients with type 1 and type 2 diabetes [[8], [9], [10]].

Endothelin-1 (ET-1) is a vasoconstrictor protein that was first identified in 1988. It is one of a family of three Endothelins that exert their action through two G-protein coupled receptors, endothelin receptor-A (ETA) and endothelin receptor- B (ETB), and ET-1. They comprise a 21-amino acid and are encoded on chromosomes 6, 1, and 20 respectively [11]. *EDN*1 gene contains 5 exons and spans ∼6.8 Kb of genomic DNA, and encodes an ET-1 protein [12]. ET-1-mediated vasoconstriction was able to prompt a hypoxic state which later stimulates pathological angiogenesis that appeared in diabetic retinopathy. The plasma ET-1 levels were found to be increased [13].

Since *EDN*1 polymorphisms and ET-1 protein have not yet been studied concerning T2DM in the Jordanian population, we hypothesized that polymorphisms in *the EDN*1 gene could be a contributing risk factor for T2DM in the Jordanian population. Also, T2DM patients could have an elevated level of ET-1 protein compared to healthy individuals. The present case-control study aimed at the investigation of the possible association between *EDN*1 rs5370 and rs2071942 G/A polymorphisms and ET-1 protein levels among patients with T2DM as compared to healthy individuals in the Jordanian population. Few studies have investigated the relationships between *EDN*1 gene polymorphisms and T2DM as well as their relation to ET-1 protein level.

## 2 Material and methods

### 2.1 Study population

Blood samples analyzed in the current study were collected from 97 confirmed T2DM patients (50 males + 47 females). The average age of the diabetic participants was 58 years. Pregnant women as well as patients with hyperinsulinemia were excluded from this study. Diagnosis of T2DM was carried out by the “National Center for Diabetes, Endocrinology and Genetics” at Jordan University/Amman. The clinical procedure was according to the American Diabetes Association (ADA) criteria.

Controls blood samples were collected from 80 diabetic-free individuals (41 males + 39 females). The average age of the control participants was 55 years. In the controls, pregnant women and any individual who has any sign of diabetes were excluded.

### 2.2 Blood sample collection

Four milliliters of venous blood were collected in EDTA-coated tubes. All samples were labeled for analysis with a specific code. The blood samples were divided into two parts 2 ml each. One part served for the separation of plasma within 2 h and the other one for DNA extraction. The blood samples were centrifuged at 1000×*g* for 20 min for separation of plasma. The separated plasma was transferred to a microcentrifuge tube and kept at −20 °C until used for the quantification of the Endothelin 1 protein using IQELIZA technique. The whole EDTA blood was kept at 4 °C to be used for DNA isolation.

### 2.3 DNA extraction

EDTA coated tubes (Greiner Bio-One), Quick-DNA™ Miniprep Kit (ZYMO RESEARCH company), 6X DNA Loading Dye, Tris-borate-EDTA 10X, RedSafe™ Nucleic Acid Staining Solution (Thermo Fisher Scientific), 5X Firepol Master Mix (Solis BioDyne OÜ) were used for DNA extraction according to the manufacturer's instructions (ZYMO RESEARCH company), where 400 μl of genomic lysis buffer was added to 100 μl of blood and mixed completely by vertexing for 4–6 s, and then kept for 5–10 min at room temperature. After that, the mixture was transferred to a Zymo-Spin™ IIC column in a collection tube, and the mixtures were centrifuged at 10,000×*g* for 1 min; the collection tube was discarded. The Zymo-Spin™ IIC column was transferred to a new collection tube, then 200 μl of DNA Pre-Wash Buffer was added to the spin column. The column was centrifuged at 10,000×*g* for 1 min. After that 500 μl of gDNA, wash buffer was added to the spin column, which was centrifuged at 10,000×*g* for 1 min. The spin column was transferred to a clean microcentrifuge tube, and ≥50 μl DNA elution buffer was added to the spin column and incubated for 2–5 min at room temperature and then centrifuged at top speed for 30 s to elute the DNA.

### 2.4 Agarose gel electrophoresis

Agarose gel was obtained from Bio basic/Canada, 1 Kb DNA ladder (Kapa biosystem/US). The isolated DNA was subjected to electrophoresis using 1.0 % agarose gel with 0.5X TBE buffer and stained with RedSafe™ Nucleic Acid Staining Solution**.** Five μl of the extracted DNA was mixed with 2 μl of 6X Kapa DNA Loading Dye for 1 h at 100 V [14] and then the gel was visualized on a short wave U.V. transilluminator.

### 2.5 Detection of *EDN1* polymorphisms rs5370

#### 2.5.1 Primer design for rs5370 polymorphisms

The sequence of the forward and reverse primers required for amplification of the region of DNA that contains rs5370 SNP (single nucleotide polymorphism) was the same, as which that was used in a Ukrainian study [15]. The primers were synthesized by Metabion International AG. (Germany)

#### 2.5.2 RFLP analysis for detection of rs5370 polymorphisms

Here, *Cac*8I restriction endonuclease (New England Biolabs, Boston MA), primers synthesized by (Metabion International AG. Germany), and *Taq*1 restriction endonuclease (New England Biolabs, Boston MA) were used. Polymerase chain reaction (PCR) was conducted by using a PCR machine (Eppendorf, Germany) to amplify the *EDN1* gene that contains the rs5370 SNP region. For each PCR reaction: 2–5 μl of genomic DNA from patients or controls were added to 4 μl of PCR master mix (5X FIREPOL Master Mix), and 0.5 μl of forward primer (0.5 μM) and 0.5 μl reverse primer (0.5 μM). The reaction mixture was topped to 20 μl by nuclease-free water. PCR conditions were as follows: 95 °C (initial denaturation) for 10 min, and 35 cycles of 95 °C for 1 min (denaturation), 61 °C for 1 min (annealing), 72 °C for 1 min and 30 s (extension) and 72 °C for 10 min (final extension). PCR products of 262 bp were digested with 0.5 μl of *Cac*81 (New England Biolabs, Boston MA) restriction endonuclease (17-h incubation) at 37 °C on a PCR machine (Eppendorf. Germany). The occurrence of guanine (G) in the 5665th location of the *EDN*1 gene led to the cleavage of the amplicon (262 bp) by *Cac*81 into two fragments of 189 and 73 bp. When the G is replaced by thymine (T), we have one intact 262 bp fragment due to the loss of the *Cac*81 restriction site. Digested products were examined on 2 % agarose gels and stained by (RedSafe™ Nucleic Acid Staining Solution). Table 1 explains the primer and PCR product length and restriction fragment length.

**Table 1:** primers sequence and PCR product size and restriction fragments size for both SNP.

Gene variants	Primer sequence	Restriction Enzyme	PCR product size	Restriction fragments size
rs5370	F: 5-TCTTGCTTTATTAGGTCGGAGACC-3 R: 5-TTTGAACGAGGACGCTGGTC-3	*Cac*81	262 bp	189 bp, 73 bp G 262 bp T
rs2071942 G/A	**F:** 5′-AAACCGATGTCCTCTGTA-3′ **R:** 5′-ACCAAACACATTTCCCTATT-3	*Taq*1	358 bp	208bp, 150bp G 358bp A

### 2.6 Detection of *EDN1* polymorphisms (rs2071942 G/A)

#### 2.6.1 Primer design for polymorphism (rs2071942 G/A)

The sequence of the forward and reverse prime required for the amplification of a region of DNA that contains rs2071942 G/A SNP was the same, as was used in a previous study (Kaňková et al., 2001). The primers were then synthesized by Metabion International AG. (Germany).

#### 2.6.2 Polymerase chain reaction-restriction fragment length

polymorphism (PCR-RFLP) for the detection of rs2071942 G/A.

PCR was carried out in a PCR to amplify the rs2071942G/A SNP region of the *EDN*1 gene. For each PCR reaction: 2–5 μl of genomic DNA from patients or control were added to 4 μl of PCR master mix and 0.5 μl of forward primer (0.5 μM) and 0.5 μl reverse primer (0.5 μM). The reaction mixture was topped to 20 μl by nuclease-free water. The following PCR amplification conditions were applied: After the initial denaturation at 94 °C for 3 min, 35 cycles were conducted: 94 °C for 30 s, annealing for 30 s, and 72 °C for 30 s. The final extension lasted for 10 min at 72 °C. rs2071942 G/A were digested with *Taq*1 restriction endonuclease (New England Biolabs, Boston MA). Digestion products include two fragments of 150 and 208 bp for the wild-type allele and 358 bp for the mutated allele. Digested products were examined on 2 % agarose gels and stained by (RedSafe™ Nucleic Acid Staining Solution). Table 1 explains the primer and PCR product length and restriction fragment length.

### 2.7 Measurement of Endothelin 1 (ET-1) level

#### 2.7.1 Measuring the level of ET-1 by Immuno Quantitative Enzyme-Linked ImumunoSorbent Assay (IQELISA)

For determining the concentration of endothelin 1 (ET-1) protein, the Immuno Quantitative Enzyme-Linked ImumunoSorbent Assay (IQELISA) was used (RayBio^R^ Human VEGF IQELISA Kit Protocol). Ninety-four plasma samples were used (52 patients and 42 controls) to measure the concentration of ET-1 protein.

For measuring the concentration of ET-1, 10–25 μl of standards and samples were added into wells, and the wells were enclosed and incubated for 2.5 h at room temperature with moderate shaking. The solutions were decanted, and the wells were washed 4 times with 1X wash solution. After the last wash, the remaining wash buffer was decanted. The plate was inverted and stained beside clean paper towels. To each well, 25 μl of detection affinity reagent was added and incubated for 1 h at room temperature with moderate shaking. The solutions were removed, and washing with 1X wash solution was repeated. Then, 25 μL of IQELISA detection reagent was added to each well. The solutions were removed, and the washing was repeated with 1X wash solution. One hundred μL of final wash buffer was added and incubated for 5 min with rocking. The solutions from each well were removed and this stage was repeated an additional two times. To each well, 100 μL of 1X PCR preparation buffer was added and incubated with rocking for 5 min before eliminating the buffer, 15 μL of the primer solution was added, and 10 μL of PCR Master Mix was added and pipetted thoroughly to mix the wells. The plate was enclosed, and placed into a real-time PCR instrument using SYBR Green compatible wavelength for detection with the following settings for cycling: 3-min activation at 95ᴼC, 10 s 95ᴼC denaturation, 25 s 55ᴼC annealing/extension, and both steps 2 and 3 were repeated 29x (RayBiotech, Inc).

#### 2.7.2 Calculation of IQELISA results

The most important information obtained from the IQELISA kit was Ct values. These values correspond to the number of cycles intended for a sample to exceed a fluorescence threshold. When the DNA is amplified extra fluorescent signal is created, through every cycle resulting in an estimated replication of the DNA. Then, elevated levels of DNA result in lower Ct values.

The mean Ct was calculated for the triplicate set of standards, controls and samples. After that, subtraction of the Ct value for each sample from the control to get the variation among the control and sample (Delta Ct), and plotting the values of the standards on a graph using a log scale for concentration on the x-axis.

The line of best-fit resolve has an equation y = mln(x) + b, where y is the Delta Ct value and x is the concentration and b from the line of best fit.

Calculation of the concentration of the unknown sample was obtained according to the following formula: (RayBio^R^ Human VEGF IQELISA Kit Protocol).

Concentration of unknown = EXP((y-b)/m)

Where y is the Delta Ct and obtained during the assay, and b and m are obtained from the line of best fit.

### 2.8 Statistical analyses

The association study was carried out using statistical analysis using the SPSS program for Windows version 22 (SPSS Inc. Illinois). Chi-square test was used to assess the existence of differences between the investigated variables. Fischer Exact test and Multinomial logistic regression were applied using the SPSS software to predict the association between *EDN*1 polymorphisms and the level of endothelin-1 with the susceptibility of diabetes mellitus in the Jordanian population. P value < 0.05 is considered an indication of significant variations among tested parameters.

## 3 Ethical considerations

The research followed the tenets of the Declaration of Helsinki. Ethical approval was obtained from the Institutional Review Board (I.R.B) of the National Center for Diabetes, Endocrinology, and Genetics (Decision No.:1/2019). The research followed the tenets of the Declaration of Helsinki. Patients and controls gave their consent for participation in the study. All of the information obtained from patients and control were kept confidential.

## 4 Results

### 4.1 Analysis of extracted genomic DNA

**Figure (1):**
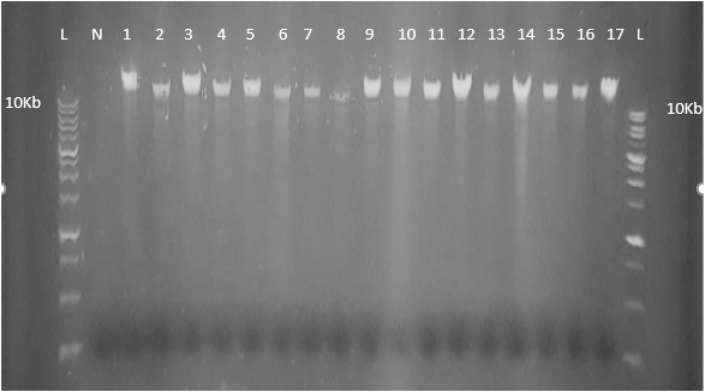
1 % Agarose gel electrophoresis of genomic DNA extracted from whole blood using (Quick-DNA™ Miniprep Kit). lanes with a symbol (L) indicated a 10 Kb DNA ladder. Lane (N) represents the negative control and numbered lanes represent the DNA samples.

Figure (1) shows that the purified genomic DNA is intact with a respectable yield in all samples and has a molecular weight larger than 10 Kb.

### 4.2 Analysis of PCR products for rs5370 SNP

**Figure (2):**
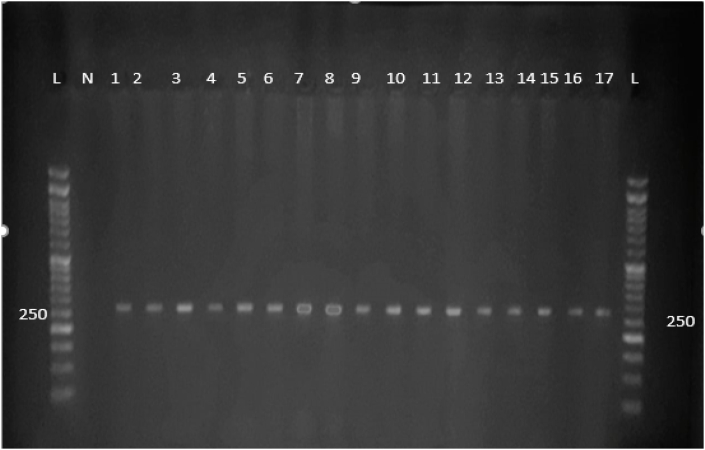
2 % Agarose gel electrophoresis of PCR products of rs5370 SNP. The figure illustrates that the fragment length (262 bp) was amplified and the intensity among bands was similar, lanes with a symbol (L) indicate the DNA ladder (100 bp DNA ladder) and lanes with a symbol (N) indicate negative controls and numbered lanes indicate the PCR products of the samples.

The purified DNA was used to amplify the rs5370 region using PCR. The amplicons (262 bp) As shown in figure (2), the amplified rs5370 variant exhibited the length of the estimated fragments of 262 bp. No primer-dimer or additional amplification was detected.

### 4.3 Analysis of PCR products for rs2071942 G/A SNP

**Figure (3):**
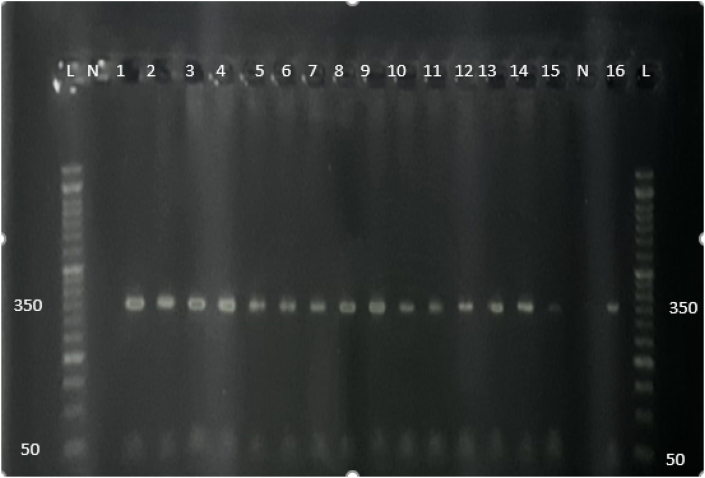
2 % Agarose gel electrophoresis of PCR products of rs2071942 G/A SNP. The figure illustrates that the fragment length (358 bp) was amplified and the intensity among bands was constant, lanes with a symbol (L) indicate the DNA ladder (100 bp DNA ladder), and lanes with a symbol (N) indicated negative controls and numbered lanes indicated the PCR products for samples.

The polymorphism of rs2071942 G/A was amplified by the PCR method. As shown in figure (3), the amplified rs2071942 G/A SNP showed bands with estimated fragments length (358 bp) and separated clearly. No additional amplification products were detected.

### 4.4 Analysis of Polymerase chain reaction-restriction fragment length polymorphism (PCR-RFLP) for rs5370 polymorphisms

**Figure (4):**
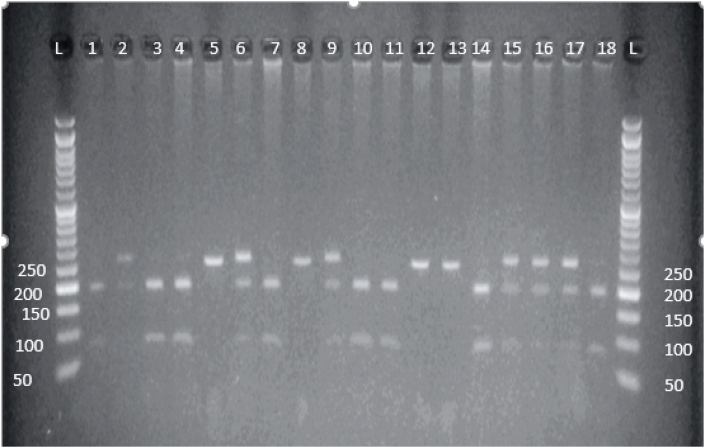
Outcome of *EDN1* rs5370 polymorphism restriction analysis.; lanes with a symbol (L) represent the DNA ladder (100 bp DNA ladder) and lanes 2, 4, 5, 8, 11,12,15, 19 GG (189 bp and 78 bp) genotype and lanes 3, 7, 10, 16, 17, 18, GT (262 bp, 189 bp, 78 bp) genotype; lanes 6, 9,13, 14 TT (262 bp) genotype.

The amplicons 262 bp (Fig. 2), were digested with 0.5 μL *Cac*81 enzyme overnight, then examined on 2 % (w/v) agarose gel. The estimated outcomes would be two fragments if the sample represents a homozygote of the normal variant (G/G), and three fragments if the sample represents a heterozygote (G/T), and one fragment is formed if the sample represents a homozygote of a polymorphic variant (T/T). Figure (4) indicated the fragments generated after incubation of PCR products for rs5370 with the enzyme.

### 4.5 Analysis of Polymerase chain reaction-restriction fragment length polymorphism (PCR-RFLP) for detection of rs2071942 G/A

**Figure (5):**
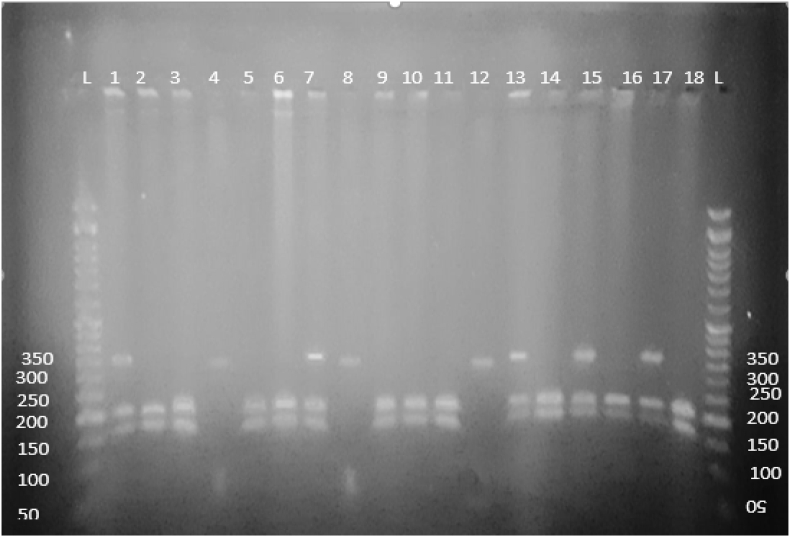
Outcomes of *EDN1* rs2071942 G/A polymorphism restriction analysis.; lanes with a symbol (L) represent the DNA ladder (100 bp DNA ladder) and lanes 2, 3, 5, 6, 9, 10, 11, 14, 18 GG genotype and lanes 1, 7, 13, 15, 17 GA genotype; lanes 4, 8, 12, TT genotype.

The amplicons (358 bp) which were obtained by PCR, were digested overnight with *Taq*1 restriction enzyme, and the products were examined on 2 % (w/v) agarose gel. Three alternative results could be obtained after the digestion of the samples. In the case of heterozygous (G/A) the size of the fragments will be 208 bp, 105 bp, and 358. Two fragments were generated when the sample was homozygous for the normal variant (G/G), 208 bp, 105 bp, and one fragment with size (358 bp) in case of a homozygous of a polymorphic variant (A/A). Figure (5) showed the fragments that were obtained after incubation of PCR products for rs2071942 G/A with restriction enzyme *Taq*1.

The demographic and physical characteristics of cases and control subjects are listed in Table 2. There were no significant differences between the two groups for age, gender, smoking status, hypertension, height, body mass index (BMI), and Endothelin1 (ET-1) protein concentration. However, there were statistically signiﬁcant differences in the weight parameter. Some parameters were observed in cases only such as neuropathy, retinopathy, and nephropathy, and cardiovascular disease.

**Table (2):** Clinical and laboratory characteristics of cases and controls.

	Cases (n (%)) 97 (54.8 %)	Controls (n (%)) 80 (45.2 %)	P-value^a^
Male	50 (51.5 %)	41 (51.2 %)	0.544
Female	47 (48.5 %)	39 (48.8 %)	1
Age (years)	58 (31–82)	55 (38–81)	0.056
Weight (kg)	84 (50–140)	77 (43–120)	0.004
Height (cm)	167(150–196)	168 (144–190)	0.081
BMI (kg/m2)	30.68 ± 5.4	27.23 ± 4.9	0.772
ET-1 (pg/ml)	52 (52 %)	42 (42 %)	0.571
Hypertension			
Yes	59 (60.8 %)	11 (13.8 %)	0.000
No	38 (39.2 %)	69 (86.3 %)	0.000
Smoking			
Yes	24 (24.7 %)	17 (21.3 %)	0.357
No	73 (75.3 %)	63 (78.8 %)	0.597
Duration of diabetes	12.51 (8–31)	–	–
Neuropathy			
Yes	22 (22.7 %)	–	–
No	75 (77.3 %)	–	–
Retinopathy			
Yes	15 (15.5 %)	–	–
No	82 (84.5 %)	–	–
Nephropathy			
Yes	9 (9.3 %)	–	–
No	88 (90.7 %)	–	–
CAD			
Yes	21 (21.6 %)	–	–
No	76 (78.4 %)	–	–

a P-value less than 0.05 is considered significant.

The genotype distribution and allele frequencies for *EDN1* gene polymorphisms rs5370, rs2071942 G/A in people with T2DM (cases) and those without T2DM (controls) are presented in Table 3. Univariate analysis didn't show signiﬁcant differences in the genotype or allele frequencies among T2DM cases as well as in controls (Table 3). The genotype distribution among cases and controls didn't signiﬁcantly diverge from the Hardy-Weinberg equilibrium, indicating that the occurrences of genotypes and alleles frequency between patients and controls are not associated with occurrences of T2DM (Table 3).

**Table (3):** Distribution of ET-1 (*EDN1*) gene rs5370, rs2071942 G/A polymorphisms genotypes and alleles between case and control.

		Cases n (%)	Controls n (%)	P value*
rs5370	Genotype GG^a^ GT^b^ TT^c^	48 (49.5 %) 44 (45.5 %) 5 (5.2 %)	48 (60 %) 30 (33.4 %) 2 (2.5 %)	0.313
	Allele frequency G T	140 (72 %) 54 (28 %)	126 (78.75 %) 34 (21.25 %)	0.154
rs2071942 G/A	Genotype GG^a^ GA^b^ AA^c^	51 (52.6 %) 41 (42.3 %) 5 (5.2 %)	51 (63.7 %) 28 (35 %) 1 (1.3 %)	0.172
	Allele frequency G A	143 (73.7 %) 51 (26.3 %)	130 (81.25 %) 29 (18.75 %)	0.06

*P-value less than 0.05 is considered significant.

a homozygous dominant.

b heterozygous.

c homozygous recessive.

The subgroup analysis by sex showed that the genotype and allele frequencies of rs5370, rs2071942 G/A polymorphisms were not significantly different between males and females (Table 4).

**Table (4):** Analysis of the association of the rs5370, rs2071942 G/A polymorphisms in males and females between case and control.

	Males n (%)	Females n (%)			
	Cases (%)	Controls (%)	Cases (%)	Controls (%)	
rs5370	Genotype	25 (50 %)	21 (51.2 %)	24 (51.1 %)	18 (46.2 %)
	GG^a^				
	GT^b^	23 (46 %)	18 (43.9 %)	21 (44.7 %)	19 (48.7 %)
	TT^c^	2 (4 %)	2 (4.9 %)	2 (4.3 %)	2 (5.1 %)
	χ2 = 0.068 P-value^a^ = 0.967	χ2 = 0.215 P-value* = 0.898			
	Allele frequency	73 (73 %)	60 (73.1 %)	69 (73.4 %)	55 (70.5 %)
	G				
	T	27 (27 %)	22 (26.9 %)	25 (26.6 %)	23 (29.5 %)
	χ2 = 0.001 P-value^a^ = 0.979	χ2 = 0.177 P-value* = 0.674			
rs2071942 G/A	Genotype	29 (58 %)	24 (66.7 %)	28 (59.6 %)	24 (61.5 %)
	GG^a^				
	GA^b^	18 (36 %)	12 (33.3 %)	19 (40.4 %)	14 (35.9 %)
	AA^c^	3 (6 %)	0 (0 %)	0 (0 %)	1 (2.6 %)
	χ2 = 2.485 P-value^a^ = 0.293	χ2 = 1.333 P-value* = 0.514			
	Allele frequency	76 (76 %)	60 (83.3 %)	75 (79.8 %)	62 (79.5 %)
	G				
	A	24 (24 %)	12 (16.7 %)	19 (20.2 %)	16 (20.5 %)
	χ2 = 1.36 P-value^a^ = 0.243	χ2 = 0.002 P-value* = 0.961			

*P-value less than 0.05 is considered significant.

a homozygous dominant.

b heterozygous.

c homozygous recessive.

Also, no association between the studied polymorphisms and T2DM was observed according to multivariate analysis adjusted for confounders between cases and controls such as age, weight, body mass index (BMI), smoking status, hypertension, blood pressure, and Endothelin1 (ET-1) protein concentration in both SNPs. Also, no association between the studied polymorphisms and T2DM was observed among cases and controls after adjusting the height and gender in the case of TT and AA alleles in both SNPs according to the codominant genetic model (Table (5), Table (6)).

**Table (5):** logistic regression analysis between the case and control for SNP rs2071942 **G/A**.

SNP_ rs2071942 GA^a^		P-value*	Odd ratio	95 % Confidence Interval for Exp(B)	
				Lower Bound	Upper Bound
GA	Age	0.142	0.976	0.946	1.008
	Height	0.029	0.947	0.901	0.994
	Weight	0.953	0.999	0.978	1.021
	Gender				
	Male	0.013	2.964	1.252	7.018
	Female				
	Hypertension				
	Yes	0.562	1.217	0.627	2.362
	No				
	Smoking				
	Yes	0.125	0.532	0.237	1.191
	No				
	ET-1	0.497	0.999	0.996	1.002
	BMI	0.507	1.241	0.656	2.346
AA	Age	0.425	0.965	0.885	1.053
	Height	0.947	0.996	0.873	1.136
	Weight	0.403	0.974	0.916	1.036
	Gender				
	Male	0.748	1.436	0.158	13.053
	Female				
	Hypertension				
	Yes	0.370	2.216	0.389	12.634
	No				
	Smoking				
	Yes	0.529	0.486	0.052	4.590
	No				
	ET-1	0.875	0.999	0.993	1.006
	BMI	0.781	0.792	0.153	4.111

*P-value less than 0.05 is considered significant.

a The reference category is GG.^b^. This parameter is set to zero because it is redundant.

**Table (6):** logistic regression analysis between the case and control for rs5370 SNP.

rs5370_SNP^a^		P-value*	Odd ratio	95 % Confidence Interval for Exp(B)	
				Lower Bound	Upper Bound
GT	Age	0.320	0.984	0.954	1.015
	Height	0.027	0.947	0.903	0.994
	Weight	0.335	1.011	0.989	1.032
	Gender Male Female	0.023	2.625	1.140	6.042
	Hypertension Yes No	0.510	1.244	0.650	2.382
	Smoking Yes No	0.930	0.966	0.449	2.078
	ET-1	0.952	1	0.997	1.003
	BMI	0.689	1.133	0.614	2.092
TT					
	Age	0.311	0.959	0.883	1.040
	Height	0.903	0.993	0.882	1.118
	Weight	0.880	0.996	0.944	1.050
	Gender				
	Male	0.683	1.535	0.196	12.016
	Female				
	Hypertension				
	Yes	0.231	2.674	0.535	13.367
	No				
	Smoking				
	Yes	0.905	1.113	0.191	6.504
	No				
	ET-1	0.958	1	0.994	1.006
	BMI	0.751	0.767	0.150	3.931

*P-value less than 0.05 is considered significant.

a The reference category is GG.

Multivariate analysis showed that the gender and height in the status heterozygous allele in both SNP showed a significant risk factor for T2DM according to the codominant genetic model (Table (5), Table (6)).

Multivariate analysis between patient groups showed that recessive allele is connected with the duration of diabetes in the rs5370 polymorphism and exhibited a significant risk for T2DM according to the codominant genetic model (Table 7). Also, no association between the studied polymorphisms and T2DM was showed after adjusting for confounders among the patients’ group such as CAD, neuropathy, retinopathy, and nephropathy according to the codominant genetic model (Table (7), Table (8)).

**Table (7):** logistic regression analysis between the patient group for rs5370 SNP.^b^

rs5370_SNP^a^		P-value*	Odd ratio	95 % Confidence Interval for Exp (B)	
				Lower Bound	Upper Bound
GT	duration diabetes	0.795	0.988	0.901	1.083
	Neuropathy				
	Yes	0.829	1.140	0.347	3.746
	No				
	Retinopathy				
	Yes	0.718	0.786	0.213	2.897
	No				
	Nephropathy				
	Yes	0.262	0.365	0.063	2.121
	No				
	CAD				
	Yes	0.183	0.466	0.151	1.435
	No				
TT	duration diabetes	0.042	1.189	1.006	1.405
	Neuropathy				
	Yes	0.998	1.949E-9	0.000	.^c^
	No				
	Retinopathy				
	Yes	0.624	1.954	.134	28.404
	No				
	Nephropathy				
	Yes	0	1.960E-8	1.960E-8	1.960E-8
	No				
	CAD				
	Yes	0.957	0.932	0.071	12.298
	No				

*P-value less than 0.05 is considered significant.

a The reference category is GG.

b . This parameter is set to zero because it is redundant.

c Floating-point overflow occurred while computing this statistic. Its value is therefore set to system missing.

**Table (8):** logistic regression analysis between the patient group for rs2071942 G/A SNP.

SNP_ rs2071942 GA^a^		P-value*	Odd ratio	95 % Confidence Interval for Exp(B)	
				Lower Bound	Upper Bound
GA	duration diabetes	0.218	0.943	0.858	1.036
	Neuropathy				
	Yes	0.595	1.385	0.417	4.607
	No				
	Retinopathy				
	Yes	0.647	1.354	0.370	4.952
	No				
	Nephropathy				
	Yes	0.150	0.267	0.044	1.614
	No				
	CAD				
	Yes	0.981	1.014	0.338	3.037
	No				
AA	duration diabetes	0.066	1.167	0.990	1.377
	Neuropathy				
	Yes	0.998	2.063E-9	0.000	.^c^
	No				
	Retinopathy				
	Yes	0.501	2.509	0.173	36.463
	No				
	Nephropathy				
	Yes	0	1.979E-8	1.979E-8	1.979E-8
	No				
	CAD				
	Yes	0.836	1.312	0.100	17.271
	No				

*P-value less than 0.05 is considered significant.

a The reference category is GG.^b^. This parameter is set to zero because it is redundant.

## 5 Discussion

The number of diabetic patients increased from 108 million in 1980 to 422 million in 2014. Prevalence is clearly noticed in low- and middle-income countries than in high-income countries. Diabetes has many consequences, including blindness, kidney failure, heart attacks, stroke, and lower limb amputation. Between 2000 and 2019, there was a 3 % increase in diabetes mortality rates by age. In 2019, 2 million deaths were attributed to diabetes and the associated kidney disease [16].

T2DM is caused by numerous genes as well as different environmental factors. Several previous studies have been carried out to assess the genetic factors associated with T2DM. New methods such as the detection of SNPs and measuring the protein concentration will lead to an improved understanding of the pathogenesis of T2DM. Also, they will enhance diagnostics, treatment, and finally prevention [4,9,17].

In the current case-control study that included 97 subjects with T2DM, and 80 healthy individuals, the determination of rs2071942 G/A, rs5370 gene polymorphisms in *EDN*1 gene was conducted by gene amplification using PCR-specific primers and then genotyping through digesting the PCR products by *Сас*81 enzyme for rs5370 G > T and *Taq*1 enzyme for rs2071942 G/A. In both SNPs, three genotypes were detected. For rs2071942 G/A: The GG wild type, GA heterozygous mutant, and AA homozygous mutant. For rs5370 G > T: The GG wild type, GT heterozygous mutant, and TT homozygous mutant. These diverse genotypes for both SNPs have different occurrences in the studied groups.

We assessed the potential effects of rs5370, and rs2071942 G/A in the expansion of T2DM in the Jordanian population through the endothelin pathway. There was no statistical significance in the genotype and allele distribution for rs5370, and rs2071942 G/A. Most of the available reports about nominated SNPs in our study for EDN1 were dedicated to rs5370, which refers to missense nonsynonymous point mutations (exon 5, Lys198Asn, chromosome position 12296022, GRCh 38.2, NCBI *Homo Sapiens* Annotation release 107). The relationship of the rs5370 with numerous diseases and phenotypes, comprising T2DM and its complication has been published. According to Ensembl genome browser 91 (exon 5, Lys198Asn, chromosome position 12296022, GRCh 38.2, NCBI *Homo Sapiens* Annotation release 107), no dangerous effects have been originating for the replacement of G by T, which lead to the transversion of Lysine (Lys) to Asparagine (Asn).

Our results concerning rs5370 are in agreement with a previous study which showed that rs5370 is not related to T2DM and its complications [18]. On the other hand, however, Li et al. (2008) recognized the mutant allele of rs5370 polymorphism as an intended risk factor for the late beginning of T2DM in addition to the complicated hazard of diabetic retinopathy in T2DM patients of Chinese lineage, and wild allele may play roles in the interruption of the blood-retinal wall in diabetic retinopathy in the T2DM [19]. Comparing the results of our study with another study that investigated the occurrences of rs5370 SNP in other diseases, it can be seen that the rs5730 polymorphic variation was connected with blood pressure in obese people of European origin [20] and Japanese lineage [21]. Another study established the influence of rs5370 polymorphism on vascular reactivity [22], whereas Treiber et al. confirmed that the rs5370 polymorphism affects blood pressure response to behavioral pressure in obese subjects from reduced socio-economic position [23].

Concerning rs2071942 G/A SNP, the results of our study are in agreement with a previous study that showed that this SNP is not associated with T2DM [24]. However, comparing our study with other studies that investigated this SNP in different cases such as investigating the differences in the genetic polymorphisms between sea level sojourners and ascent to high altitudes. It was shown that the polymorphisms in *EDN*1 9127 G/A, were detected to be significantly unrelated among the Ladakh natives and the sea level acclimatized sojourners. The occurrences of the wild allele of rs2071942 G/A were significantly greater in the Ladakh natives related to sea level acclimatized sojourners. The recessive allele has minor advantages throughout acclimatization when compared to the wild allele [24]. The Sea-level sojourners undertake accommodation through combined physiological processes for protecting the body alongside oxygen deficiency while the high-altitude natives (inhabitant population) are adjusted to the predominant hypobaric hypoxic situation by natural selection. The intronic variant at intron 4 (G/A, chromosome position 12294760, GRCh 38.2, NCBI *Homo Sapiens* Annotation release 107) has been described to be restricted linkage disequilibrium (LD) through an efficient exonic SNP, rs5370 (exon 5, Lys198Asn, chromosome position 12296022, GRCh 38.2, NCBI *Homo Sapiens* Annotation release 107). The mutant allele is linked to cardiomyopathy when compared to the protective outcome of the wild allele [25].

The results of our study are in disagreement with previous studies which showed that height and gender did not represent a significant risk factor for occurrences of the heterozygous allele in both SNP. Some studies indicated that the duration of diabetes did not represent a risk factor for occurrences of the recessive allele in rs5370 [[18], [19], [20], [21], [22], [23], [24],^26^]. We believe that the disagreement between our results and those of previous studies is due to the small sample size in our study.

Furthermore, the results of our study regarding ET-1 concentration revealed that the concentration of ET-1 didn't have a statistical significance difference between diabetic patients and healthy individuals, and this is in disagreement with previous studies that indicated increased ET-1 concentrations in patients with diabetes mellitus [27]. Another investigation showed an elevated level of ET-1 in type 2 diabetic Goto-Kakizaki (GK) rats. Based on these results they have suggested that the elevated concentration of ET-1 is related to diabetic endothelial cell damage, which plays an important role in diabetic vascular complications [28].

Concerning the studies that correlate the *EDN*1 polymorphisms with plasma ET-1 concentration, it has been shown that in Chinese patients with diabetic nephropathy, the recessive allele A of *EDN*1 was connected with reduced plasma proendothelin-1 level [18]. An additional study indicated that the substitution of G by T, which lead to the replacement of Lys by Asn, was connected to the increase in the levels of plasma C-terminal-pro-endothelin-1, in people of European lineage [29]. The study was carried out to realize the alteration in the genetic polymorphisms among sea-level sojourners and ascent to high altitudes. The high altitude has decreased plasma levels of endothelin; it would be relevant that the raised levels of endothelin and greater expression of endothelin converting enzyme (*ECE*1) originated in lowland individuals who established high altitude pulmonary edema. While no differences in gene expression were distinguished between both groups of people [26].

However, taking the following results of the present work into consideration.

1 that recessive allele is connected with the duration of diabetes in the rs5370 polymorphism and exhibited a significant risk for T2DM according to the codominant genetic model,
2 height and gender showed to be significant risk factors in the presence of heterozygote alleles in both SNPs, rs2071942 G/A and rs5370 SNPs,

we can suggest that such SNP's may be utilized as an indication for prognosis, predisposition screening as well as in the genome-wide association studies on T2DM. Taking all currently studied SNP's and any others in the future, reaching a genome-wide association study on T2DM may help enhance the clinician's ability to effectively manage T2DM and improve patient outcomes through the followings.

1 Treatment Tailoring: The findings may shed light on specific subgroups of T2DM patients who may respond better to certain treatment modalities. This information can help clinicians personalize treatment plans and optimize therapy choices based on individual patient characteristics, such as genetic factors or metabolic profiles.

2- Risk Assessment and Early Detection: The findings may identify novel biomarkers or risk factors associated with T2DM complications or disease progression. By incorporating these markers into risk assessment models, clinicians can identify high-risk patients at an earlier stage and intervene promptly to prevent or at least delay complications.

3 Precision Medicine Approaches: The findings may support the development of precision medicine approaches in T2DM management. Clinicians can use the knowledge gained from these findings to tailor treatment strategies, including medication selection, dosing, and timing, based on a patient's unique genetic profile or molecular characteristics.
4 Lifestyle Modifications: The findings may provide evidence-based recommendations on lifestyle modifications, such as diet and exercise regimens, that have demonstrated efficacy in managing T2DM. Clinicians can use this information to educate and guide patients towards adopting healthier lifestyle habits, improving glycemic control, and reducing the risk of complications.
5 Patient Education and Empowerment: Incorporating these findings into patient education programs can enhance patient understanding of T2DM and its management. Clinicians can effectively communicate the rationale behind treatment decisions, empower patients to actively participate in their care, and promote adherence to treatment plans.

However, it is important to note that the implementation of these findings into clinical practice should be done in a thoughtful and evidence-based manner. Further research, validation, and consideration of individual patient characteristics are essential to ensure safe and effective application of these and any other findings in the management of T2DM.

## 6 Conclusions

This study is an extension of the studies that examined the effect of the *EDN*1 polymorphisms and ET-1 protein on T2DM. To our knowledge, this is the first study that examined the effect of *EDN*1 polymorphisms and ET-1 protein on T2DM in the Middle Eastern population, particularly in Jordan.

The current study reported four findings: There was no significant association between *EDN*1 genotypes between T2DM and healthy individuals, and there was no significant association of ET-1 protein level between T2DM and healthy individuals. Moreover, there was a significant association between height and gender and occurrences of the heterozygous allele for rs2071942 G/A and rs5370 SNPs, and finally, there was a significant association between the duration of diabetes and occurrences of the recessive allele for rs5370. The disagreement between our results and other genetic association studies may be relatively clarified by the dissimilarity in the genetic or environmental factors of the populations considered, or by the small sample size.

## Data availability

All data generated or analyzed during this study are included in this published article.

## CRediT authorship contribution statement

**Ahmed O. Maslat:** Writing – review & editing, Writing – original draft, Supervision, Project administration, Methodology, Funding acquisition, Formal analysis, Data curation, Conceptualization. **Omar M. Al-Mahmood:** Writing – review & editing, Writing – original draft, Visualization, Software, Methodology, Data curation, Conceptualization. **Nahla M. Al Khawaja:** Writing – review & editing, Writing – original draft, Validation, Data curation, Conceptualization. **Ramadan Al-Shdefat:** Writing – review & editing, Writing – original draft, Methodology, Formal analysis, Data curation, Conceptualization.

## Declaration of competing interest

The authors report no conflict of interest associated with this manuscript.

## References

[1] At K., Hm D. (2015). Diabetes mellitus: the epidemic of the century. World J. Diabetes.

[2] Sargazi S., Ravanbakhsh M., Nia M.H., Mirinejad S., Sheervalilou R., Majidpour M., Danesh H., Saravani R. (2022). Association of polymorphisms within hox transcript antisense rna (hotair) with type 2 diabetes mellitus and laboratory characteristics: a preliminary case-control study. Dis. Markers.

[3] Galavi H., Noorzehi N., Saravani R., Sargazi S., Mollashahee-Kohkan F., Shahraki H. (2018). Genetic polymorphism in ADRB-1 is associated with type 2 diabetes susceptibility in Iranian population. Gene Reports.

[4] Sadeghi M.B., Nakhaee A., Saravani R., Sadeghi M.H., Sargazi S., Nia M.H. (2021). SIRT1 functional polymorphisms (rs12778366, rs3758391) as genetic biomarkers of susceptibility to type 2 diabetes mellitus in Iranians: a case-control study and computational analysis. Int. J. Diabetes Dev. Ctries..

[5] Asmat U., Abad K., Ismail K. (2016). Diabetes mellitus and oxidative stress—a concise review. Saudi Pharmaceut. J..

[6] Skyler J.S. (2009). Intensive glycemic control and the prevention of cardiovascular events: implications of the accord, advance, and va diabetes trials: a position statement of the American diabetes association and a scientific statement of the American college of cardiology foundation and the American heart AssociationResponse to lund and vaag. Diabetes Care.

[7] Versari D., Daghini E., Virdis A., Ghiadoni L., Taddei S. (2009). Endothelial dysfunction as a target for prevention of cardiovascular disease. Diabetes Care.

[8] Sadeghi M.B., Nakhaee A., Saravani R., Sargazi S. (2021). Significant association of LXRβ (NR1H2) polymorphisms (rs28514894, rs2303044) with type 2 diabetes mellitus and laboratory characteristics. J. Diabetes Metab. Disord..

[9] Sargazi S., Heidari Nia M., Sargazi F.M., Sheervalilou R., Saravani R., Mirinejad S. (2020). SNPs in the 3’-untranslated region of SLC30A8 confer risk of type 2 diabetes mellitus in a south-east Iranian population: evidences from case-control and bioinformatics studies. J. Diabetes Metab. Disord..

[10] Naruse M., Naruse K., Demura H., Nakamura N., Kubo K., Kato M., Sugino N., Hagiwara H. (1992). Plasma immunoreactive endothelin levels are increased in hemodialysis patients with hypertension following erythropoietin therapy. Hypertens. Res..

[11] Fyhrquist F., Saijonmaa O., Metsärinne K., Tikkanen I., Rosenlöf K., Tikkanen T. (1990). Raised plasma endothelin-I concentration following cold pressor test. Biochem. Biophys. Res. Commun..

[12] Stow L.R., Jacobs M.E., Wingo C.S., Cain B.D. (2011). Endothelin-1 gene regulation. Faseb. J..

[13] Kawamura M., Ohgawara H., Naruse M., Suzuki N., Iwasaki N., Naruse K., Hori S., Demura H., Omori Y. (1992). Increased plasma endothelin in NIDDM patients with retinopathy. Diabetes Care.

[14] Khalil R., Al-Awaida W.J., Al-Ameer H.J., Jarrar Y., Imraish A., Al Bawareed O., Qawadri R., Al Madhoon F., Obeidat L. (2020). Investigation of ACE rs4646994, MTHFR rs1801133 and VDR rs2228570 genotypes in Jordanian patients with fibromyalgia syndrome. Endocr. Metab. Immune Disord. - Drug Targets.

[15] Dubovyk Y.I., Oleshko T.B., Harbuzova V.Y., Ataman A.V. (2018). Positive association between EDN1 rs5370 (Lys198Asn) polymorphism and large artery stroke in a Ukrainian population. Dis. Markers.

[16] W. report; 5 A. 2023, Diabetes, WHO Report; 5 April 2023. (n.d.). https://www.who.int/news-room/fact-sheets/detail/diabetes.

[17] Van Tilburg J., Van Haeften T.W., Pearson P., Wijmenga C. (2001). Defining the genetic contribution of type 2 diabetes mellitus. J. Med. Genet..

[18] Bregar D. (2018).

[19] Janice W., Louey C., Kwong Wai Choy, Liu David T.L., Wai Man Chan, Yiu Man Chan, Nicholas S K Fung, Bao Jian, Fan, Baum Larry, Chan Juliana C.N., Lam Dennis S.C. (2008). EDN1 Lys198Asn is associated with diabetic retinopathy in type 2 diabetes. Mol. Vis..

[20] Tiret L., Poirier O., Hallet V., McDonagh T.A., Morrison C., McMurray J.J.V., Dargie H.J., Arveiler D., Ruidavets J.B., Luc G., Evans A., Cambien F. (1999). The Lys198Asn polymorphism in the endothelin-1 gene is associated with blood pressure in overweight people. Hypertension.

[21] Asai T., Ohkubo T., Katsuya T., Higaki J., Fu Y., Fukuda M., Hozawa A., Matsubara M., Kitaoka H., Tsuji I., Araki T., Satoh H., Hisamichi S., Imai Y., Ogihara T. (2001). Endothelin-1 gene variant associates with blood pressure in obese Japanese subjects: the Ohasama study. Hypertension.

[22] Iglarz M., Benessiano J., Philip I., Vuillaumier-Barrot S., Lasocki S., Hvass U., Durand G., Desmonts J.M., Lévy B.I., Henrion D. (2002). Preproendothelin-1 gene polymorphism is related to a change in vascular reactivity in the human mammary artery in vitro. Hypertension (New York).

[23] Treiber F.A., Barbeau P., Harshfield G., Kang H.S., Pollock D.M., Pollock J.S., Snieder H. (2003). Endothelin-1 gene LYS198ASN polymorphism and blood pressure reactivity. Hypertension.

[24] Kaňková K., Mužík J., Karásková J., Beránek M., Hájek D., Znojil V., Vlková E., Vácha J. (2001). Duration of non-insulin-dependent diabetes mellitus and the TNF-β NcoI genotype as predictive factors in proliferative diabetic retinopathy. Ophthalmologica.

[25] Taylor M.R.G., Slavov D., Humphrey K., Zhao L., Cockroft J., Zhu X., Lavori P., Bristow M.R., Mestroni L., Lazzeroni L.C. (2009). Pharmacogenetic effect of an endothelin-1 haplotype on response to bucindolol therapy in chronic heart failure. Pharmacogenetics Genom..

[26] Tomar A., Malhotra S., Sarkar S. (2015). Polymorphism profiling of nine high altitude relevant candidate gene loci in acclimatized sojourners and adapted natives. BMC Genet..

[27] Schneider J.G., Tilly N., Hierl T., Sommer U., Hamann A., Dugi K., Leidig-Bruckner G., Kasperk C. (2002). Elevated plasma endothelin-1 levels in diabetes mellitus. Am. J. Hypertens..

[28] Harris A.K., Hutchinson J.R., Sachidanandam K., Johnson M.H., Dorrance A.M., Stepp D.W., Fagan S.C., Ergul A. (2005). Type 2 diabetes causes remodeling of cerebrovasculature via differential regulation of matrix metalloproteinases and collagen synthesis: role of endothelin-1. Diabetes.

[29] Verweij N., Mahmud H., Leach I.M., De Boer R.A., Brouwers F.P., Yu H., Asselbergs F.W., Struck J., Bakker S.J.L., Gansevoort R.T., Munroe P.B., Hillege H.L., Van Veldhuisen D.J., Van Gilst W.H., Silljé H.H.W., Van Der Harst P. (2013). Genome-wide association study on plasma levels of midregional- proadrenomedullin and C-terminal-pro-endothelin-1. Hypertension.

